# “It Is Like Medicine”: Using Sports to Promote Adult Women’s Health in Rural Kenya

**DOI:** 10.3390/ijerph18052347

**Published:** 2021-02-27

**Authors:** Francis Barchi, Millan A. AbiNader, Samantha C. Winter, Lena M. Obara, Daniel Mbogo, Bendettah M. Thomas, Brittany Ammerman

**Affiliations:** 1 Edward J. Bloustein School of Planning & Public Policy, Rutgers, The State University of New Jersey, New Brunswick, NJ 08901, USA; 2 Office of Gender-Based Violence, School of Social Work, Arizona State University, Phoenix, AZ 85004, USA; Millan.AbiNader@asu.edu; 3 School of Social Work, Columbia University, New York, NY 10027, USA; scw2154@columbia.edu; 4 School of Social Work, Rutgers, The State University of New Jersey, New Brunswick, NJ 08901, USA; moraaobara@gmail.com; 5 Village Voices, Kerugoya 10300, Kenya; kindaniel@ymail.com; 6 Nikumbuke Project, Lunga 80402, Kenya; bendettah@gmail.com; 7 Pennsylvania State University College of Medicine, Hershey, PA 17033, USA; briammerman10@yahoo.com

**Keywords:** women’s health, sports, non-communicable disease, obesity, physical activity, health promotion, mixed-methods, Africa

## Abstract

Despite the well-documented health benefits of recreational sports, few opportunities exist in lower- and middle-income countries for adult women to participate in recreational physical activities. An explanatory sequential mixed methods approach was used to explore associations between an innovative soccer program for adult women and self-reported health status. Cross-sectional survey data were collected in 2018–2019 from 702 women in the Nikumbuke Project, a health and literacy program in southeastern rural Kenya, followed by focus group discussions with 225 women who also participated in the Project’s soccer program. Quantitative findings suggest that women who participated in soccer had 67% greater odds of reporting good or excellent health than their non-soccer playing peers. Thematic analysis of qualitative data indicated that women credited soccer with less pain, fatigue, and stress, as well as weight loss and reduced dependence on medicine for hypertension, pain, and sleep problems. Women equated health benefits with greater ease and efficiency in completing chores, reduced worries, youthful energy, male-like strength, and pleased husbands. Soccer programs for adult women may be particularly effective interventions in settings where access to health care is limited and where lack of opportunity to engage in physical aerobic activity increases women’s risks for poor health outcomes.

## 1. Introduction

Goal 3 of the Sustainable Development Goals calls for the reduction of non-communicable disease (NCD)-related mortality by one-third within the next decade [1]. Epidemiologic data suggest that this target is attainable only if major reductions in NCD-related mortality in low- and middle-income countries (LMIC), which bear as much as 80% of the NCD burden, are achieved [2]. While rates of key NCD-risk behaviors such as tobacco and harmful alcohol use are higher in males than females, women are at greater risk of being overweight/obese, and, in LMIC countries, the rate of obesity is increasing at a faster rate in rural areas than urban ones [3]. Adiposity is a driver of the rising rate of diabetes across Africa, with women at greater risk of mortality from the disease than men [4,5]. In Kenya, 38.5% of women aged 18–44 years are estimated to have a Body Mass Index (BMI) in excess of 25 (overweight) and rates of obesity in women increase across the lifespan [6]. 

The burden of NCDs in Kenya is on the rise, with NCDs accounting for 27% of all deaths and 50% of all hospital admissions in 2015 [7]. This increase in NCD risk is attributable in part to a decrease in aerobic physical activity in the population. Reversing this trend has been identified as a central objective in the country’s national strategy for combatting NCDs [8]. Among women in Sub-Saharan Africa, physical inactivity has been linked to overweight/obesity, hypertension, and diabetes among women [9,10,11,12,13]. 

Although most women in rural settings engage in considerable physical activity revolving around domestic chores, food production, child care, and the fetching of water, they have limited opportunity to engage in recreational sports [8]. This is often due to a lack of material resources, cultural norms discouraging the wearing of tight-fitting clothes when participating in sports, and the gendered nature of domestic and household chores that afford women limited time for leisure activities of any kind [9,14]. As such, women may have limited access to physical activities that offer aerobic exercise or provide the social and mental health benefits of team sports. While opportunities exist in the region for men to participate in sports such as soccer and rugby, sport remains a gendered domain that precludes adult women from participating in organized recreational physical activities [10]. Programs that provide adult women with opportunities for aerobic physical activity could yield positive physical and mental health benefits and reduce women’s risks of NCDs across the lifespan.

Despite the growing burden of NCDs in East Africa and the adoption of healthy lifestyles as a risk-reduction strategy in countries like Kenya, almost no research has been conducted to date on adult women’s engagement in organized recreational sports nor on the effects of such activity on women’s physical and mental health. This study was undertaken to address this gap by examining the relationship between women’s health and their participation in a recreational soccer league in rural Kenya. We hypothesized that women’s participation in the soccer league would be positively associated with women’s self-reported health status. 

## 2. Materials and Methods

This study used an explanatory sequential mixed methods approach to examine the relationship between women’s participation in an adult women’s soccer league in rural Kenya and various self-reported measures of physical and mental health [15]. In the first phase, quantitative methods were used to identify the correlates of self-reported health status in a population of adult women belonging to a health and literacy program in which a soccer league is embedded. Findings from cross-sectional survey data guided the development of a follow-on qualitative phase in which a subset of the sample was purposively recruited to provide a more nuanced understanding of the relationship between health status and soccer participation. The study took place between August 2018 and March 2019. All women participating in the study provided oral consent.

### 2.1. Setting

Lunga Lunga is a largely inland sub-county of Kwale County in southeastern Kenya. Most residents subsist on farming and herding, income-generation through informal markets, or remits through labor migration to more urban areas in the county [16]. There are high rates of poverty, food insecurity, and unemployment; formal transportation and communication infrastructure is limited. Health services are provided at one small referral hospital, two health centers (both of which are private), and 21 dispensaries which are generally staffed by a nurse or community health worker [17]. Despite national commitments to universal health coverage, only 28.5% of the population in Kwale County has health insurance [18]; most families pay their medical expenses out of pocket, resulting in low health-service utilization rates and limited uptake of preventive care. In 2018, only one-tenth of the county population had been screened for NCDs [16]. 

The Nikumbuke Project, in which this study is situated, is a Kenyan non-governmental organization (NGO) established in 2012 and supported by a Swedish nonprofit, From One to Another, and US-based Health by Motorbike [19]. The NGO provides basic literacy and health promotion services for its adult female members and school-fee subsidies for their daughters. At the time of this study, the communities participating in the Nikumbuke Project included seven settled villages, a Maasai community, one mixed Maasai/settled village, and Lunga Lunga, a market town and the sub-county seat. These ten communities, which reflect a diversity of religions as well as ethnicities, are located across the sub-county, with some as far away as 40 km from the Nikumbuke headquarters in Lunga Lunga. While several villages are situated along the major transportation route for goods between Mombasa, Kenya’s major port city, and Dar es Salaam in Tanzania, most roads within and between villages are unimproved dirt paths. Travel between villages is mostly on foot or motorbike, and paths often become impassable for all but foot traffic during the rainy seasons. 

In 2014, at the request of the Nikumbuke membership, a US-based nonprofit, the Nikumbuke Soccer League (NSL), was created to build and support an all-women’s adult soccer league, the first of its kind in Kenya. At the time of the study, each of the ten villages had organized a team of 22–23 players. While all soccer players are members of the Nikumbuke Project, not all project members participate in the soccer league. The teams practiced by themselves each week except during planting, harvesting, and the rainy season, with monthly inter-village matches and an annual tournament. 

### 2.2. Quantitative Phase

A semi-structured interview instrument was developed to collect demographic information from all members of the Nikumbuke Project as well as information about their roles and responsibilities, gender attitudes, relationships, general health, and daily activities. Eighteen women nominated by the Nikumbuke membership to assist in data collection were invited to help develop the survey questions, participate in a week-long training program in survey administration and research ethics, and work in the field under the direction of two members of the study team (Authors 3 and 5). Data collectors received a small incentive for successfully completing the training program and for each survey administered. The surveys took between 35 to 65 min to complete and were conducted in private settings. Surveys were available in both English and Kiswahili (the two official languages of Kenya); data collectors in the two communities that were home to Maasai were also able to translate questions into Maa (the language of the Maasai) as needed. Women who agreed to participate in the survey were also thanked with a small incentive.

#### 2.2.1. Sample

Women who were members of the Nikumbuke Project at the time of data collection, eighteen years or older, and able to provide verbal informed consent were eligible to participate in the survey. Although the data collectors each completed surveys as part of their training, these surveys were not included in the analytic sample. Of the 702 eligible women, there were no refusals and after excluding the data field team, the final analytic sample included 684 women.

#### 2.2.2. Measures

A series of questions were adapted from the Medical Outcomes Study survey instrument [20]. The outcome variable of interest was self-reported health status (SRHS), measured by women’s responses to a five-level question, “In general, would you say your health is excellent, very good, good, fair, or poor?” Responses to this question were collapsed into a dichotomous variable with values “poor or fair” and “good, very good, or excellent”. Five additional health-related questions were included. Women were asked three questions assessing the extent to which pain, physical health, or emotional health may have interfered with or limited their daily work or social activities in the past four weeks, and two additional questions asked if, during the same time-period, they had had a lot of energy or felt downhearted and blue. 

Sociodemographic measures were also included to record age level, number of children, relationship status, education, employment, village, and household headship. Based on reported village of residence, a variable “type of village” was created to indicate whether the respondent resided in the Maasai community, a settled village, the mixed Maasai/settled community, or the market town. Women were also asked to indicate whether there was a health clinic/dispensary in their community. Women identified as NSL members were also asked if they had played on their community’s league team for at least one season. 

#### 2.2.3. Data Analysis Plan

Data analyses were conducted using STATA16 [21]. Frequencies were run for all variables and Pearson’s chi-square tests of independence examined their association with SRHS. A logistic regression model including all variables found to be significantly associated with SRHS at the bivariate level was run to examine the relationship between women’s participation in the soccer program and SRHS. The percentage of missing values ranged from 0 to 9.5% for age, and only 86% of the 684 women had complete data for analysis. Data were primarily missing due to item non-response. We generated 14 multiply imputed (MI) datasets using Stata 16’s “mi impute chained” command. As recommended by White and colleagues, the number of imputations was determined by the percentage of incomplete cases due to missingness [22]. Analyses run on each dataset were pooled according to Rubin’s rules [23], and imputed values were found to compare reasonably with observed values. The results from the logistic regression analysis using MI were similar in magnitude and direction to those using listwise deletion; MI results are reported in this article.

### 2.3. Qualitative Phase

The study team utilized findings from the quantitative phase to shape the design, sample, and focus of the qualitative phase. The quantitative data suggested a significant positive relationship between a woman’s participation in the soccer program and how she viewed her health. For the qualitative phase, a purposeful sample of women from the Nikumbuke Project who played in the NSL (*n* = 229) were invited to participate in focus group discussions (FGD) to explore this relationship further. Of these women, 225 women agreed to participate.

At the start of each FGD, Authors 3 and 4 explained the purpose and structure of the FGD and asked for women’s oral consent to participate and be video-recorded for transcription. Nikumbuke members who had served as data collectors during the quantitative phase of the study were invited to receive training and assist in the coordination of the discussions. Training sessions were held to review study goals and objectives, qualitative data collection, and ethical issues. Small financial stipends were made to the data field team for their assistance in coordinating the qualitative phase; FGD participants received a small incentive as thanks for their time.

Twenty focus groups with NSL members, two per village team, were facilitated by Authors 3 and 4 in February and March 2019. Each focus group included 10–13 members of the same team and lasted approximately 90 min. Sessions followed a script containing open-ended questions about women’s motivations in joining the soccer league and the ways in which playing on the team had affected their general health, their relationships, and their roles in the community. Eighteen of the FGDs were conducted in Kiswahili and two in Maa. Video recordings of sessions conducted in Kiswahili were initially transcribed into Kiswahili and then translated into English by a transcription service in Nairobi. The two sessions conducted in Maa were simultaneously transcribed and translated directly into English by a language instructor in Nairobi fluent in Maa, Kiswahili, and English. An error in the audiotaping identified at the start of thematic coding necessitated the omission of two focus group sessions for this analysis, resulting in an analytic sample of data from 18 focus groups involving 201 women who played in the NSL. 

#### Analysis Plan 

The qualitative phase used a thematic analysis approach to identify and interpret patterns in the data [24]. Authors 1 and 2 conducted the qualitative analysis; both authors have experience at the study site, but neither facilitated the FGDs. After familiarizing themselves with the data by reviewing each transcript, the two authors independently drafted a list of initial codes and then met to generate a common set of codes. Next, each reader coded two focus groups independently and then compared coding choices in order to refine the codebook. Using this revised codebook, each reader independently coded six FGDs to assess interrater reliability. The interrater reliability (Cohen’s kappa) was, on average, 97% (range 94–99%). The two readers then met to align code definitions after which Author 2 independently finished coding the remaining FGDs, discussing issues as needed. After coding, the two met to refine the codes into themes and subthemes. The focus of this paper is the health-related themes. This collaborative approach helped to ensure interrater reliability and rigor. Qualitative findings were then shared with the remaining five authors, including those authors who had facilitated the focus groups [Authors 3 and 4]; all authors collaborated on the interpretation of the findings. Quoted passages reported in the findings were identified by a code reflecting the village team, the particular FGD within that team, and respondent number assigned in transcription to the speaker.

## 3. Results

### 3.1. Quantitative Findings

Descriptive statistics and bivariate analysis results are reported in Table 1. The majority of respondents were between the ages of 25 and 49, were married with 3 to 5 children, had little or no primary education, had not worked for wages in the past year, and were not the heads of their households. Most women had no clinic in their communities, lived in settled villages, and did not play in the NSL. Nearly 60% of women characterized their health as being good or excellent. Although the majority of respondents reported that their physical and emotional health had not recently interfered with their work responsibilities or social activities, a notable minority indicated that their health had interfered some of the time to all of the time with their normal work (29%), moderate activities (45%), and social activities (26%). Over one third reported that they had felt blue or downhearted some of the time or all of the time in the past month. All variables of interest, with the exception of clinic location, were significantly correlated with self-reported health status.

In the logistic regression, soccer team participation, age, type of village, number of children, education, effects of pain on normal work, effects of health on moderate activities, and emotional health were found to be significant predictors of SRHS, controlling for covariates (see Table 2). Women who played in the NSL had 67% greater odds of reporting good to excellent SRHS than their non-soccer-playing peers (OR 1.67, CI95 1.08, 2.57, *p* = 0.021). Women between the ages of 25 and 34 had 1.9 times the odds of a woman aged 35 to 49 years that they would report good to excellent SRHS (OR 1.86, CI95 1.07, 3.24, *p* = 0.028). Women with only one to two children were 2.4 times less likely to report good to excellent SRHS than women with three to five children (OR 2.37, CI95 1.14, 4.94, *p* = 0.021). Women who had completed secondary school or higher had lower odds of reporting good health than women with no education (OR 0.37, CI95 0.15, 0.90, *p* = 0.028). Women who reported that pain or general health in the past four weeks had limited their work and/or ability to do moderate activities were significantly less likely to report that their health was good or better than women without these complaints. Similarly, women who reported having felt downhearted or blue a good bit of the time or all of the time had 54% lower odds of reporting good or better health than women who reported little to no such difficulty in the past 4 weeks (OR 0.54, CI95 0.34, 0.86, *p* = 0.009).

### 3.2. Qualitative Findings

**Figure 1:**
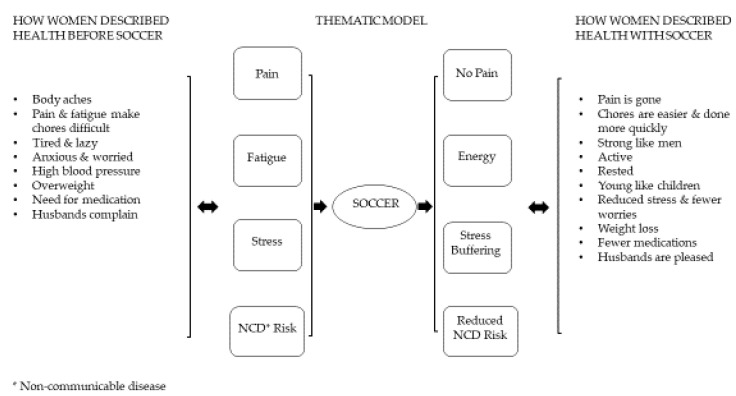
Thematic model derived from focus group discussions.

Four main themes emerged during the FGDs as women talked about their health before and after they began to play soccer: pain, fatigue, stress, and non-communicable disease (NCD) risk. These themes emerged in how the women described soccer’s effect on their ability to undertake daily responsibilities for household production and to meet their husbands’ expectations of them as wives and helpmates (see Figure 1). Women used the terms “soccer” and “football” interchangeably throughout their discussions.

#### 3.2.1. “*I Can Feel the Difference, No More Back Pains*” (KIW1-R2)

Women reported that before joining the soccer team they had been “having body aches” (GOW1-R4), “a lot of pain in my thighs” (JIW2-R3), and “a lot of back pains, even bending and turning was a problem” (KIW1-R2). Many viewed soccer as a way to reduce pain: “we were told it was best to go stretch in the field”. When women first joined, they found it was painful, commenting that

When I started playing football, I used to feel pain in my leg, but when I continued playing I am grateful that I stopped feeling the pain. I had waist complications, my husband would complain every time when I am not good in bed. When, however, I continued playing football, I am grateful, he does not complain any more.

(PEW2-R3)

Start-up pains aside, women described how playing soccer had eliminated their regular pain and how this, in turn, had made their chores easier and faster to do.

Before I could not even turn in bed when I am sleeping. When I go to fetch water, I would come back with back pains but when I started this exercise, even if you tell me to go fetch ten buckets I do not see a problem. Even my husband says that since I started playing soccer, I finish all my work fast.

(JIW2-R1)

Women talked often about the pain they felt as they went about their daily lives both in terms of their chores and their intimate relations. After they joined and adjusted to playing soccer, the pain went away; chores became easier and could be completed more quickly, and sexual relations with their partners improved.

#### 3.2.2. “*I No Longer Feel Sleepy Because Playing Gave Me Strength.*” (MAW1-R7)

Women spoke frequently about soccer increasing their ability to accomplish their tasks, describing how they went from being tired to feeling energized, strong, “light”, and younger. Often women characterized the change in their energy levels in terms of a new ability to complete domestic tasks without tiring, observing that they now were able to “walk to and from the market” (MPW2-R1), “take the goats to eat” (JUW1-R6), or “fetch water to do laundry” (JIW12-R1). 

Other women spoke of their increased energy in terms of having become strong:

Having joined the team, I feel strong and able to do my work much more easily. I used to sleep all the time and now I can do my work. For example, ploughing my gardens, milking cows, and fetching firewood without getting tired easily. Even when walking I feel good.

(MAW1-R3)

Women also described their transformation from feeling tired to being strong, a trait they perceived as one belonging to men. If women played with strength, one participant observed, “you will think we are men” (JIW2-R6). This strength made them physically equal to men on the field as well as physically equal at home:

…we play football just the same way [men] do. If it is getting into the field and running, we do the same. We kick the ball just as they do. They compete in tournaments as we do so I think it is just the same. Secondly when I leave the field and go home, there is no chore that I cannot do, so we are the same.

(MGW1-R3)

Women further described their newfound strength and energy using metaphors of lightness and youth. Women referred to their bodies as becoming “lighter” as a way to indicate more energy and newfound agility. As one woman put it,

Before I started playing soccer, my body was quite heavy. I would get tired from walking very short distances. It is as though I was very old. I was not even able to do some house chores because I would get tired very fast. But after I joined, I felt my body was lighter. Even if I wake up at four in the morning and sleep at 11 at night my body can handle it. Playing soccer has really helped us to be quick; there is no work we can say we are unable to do.

(GOW2-R8)

Additionally, women frequently remarked that having more energy and less fatigue made them feel and look young again, expressed in comments such as “when you see me playing in the field, you would think I am a young girl but I’m old” (GOW2-R10).

When you play football, you do not age. We even went to play somewhere to compete, and some of us here were referred to as girls. The truth is that some have three or four children.

(JUW1-R6)

Before joining a team, women reported that they had limited energy for their chores, tired easily, and, on occasion, displeased their husbands. Now they had vitality, were proud of their abilities to do chores, glad that their husbands were happy, and pleased to be seen as “young women”. Women saw a direct, causal relationship between soccer and this new energy, strength, and youthfulness.

#### 3.2.3. “*It Has Helped Us Because a Woman Leaves Her Home with a Lot in Her Head but When She Mixes with the Others, the Stress Goes Away.*” (LUW2-R4)

Women spoke frequently about their worries at home. They shared that, before joining the soccer league, worries had accumulated, a problem that was alleviated by soccer:

When you stay at home, you have a lot of thoughts. When you fight with your husband, you become so stressed. If you do not have anything to do, you start thinking about the fight you had with your husband and as a result you become more stressed. But when you get to the field and play with your friends, you forget all that. Staying at home you become stressed because you are idle, but when you are playing you become like a small child and you have fun.

(JIW1-R8)

Soccer was widely seen as a release from daily stressors, whether it was fighting with one’s husband or “having thoughts about what my children will eat” (LUW2-R7). Even when women arrived at practice with lots of worries, these “thoughts disappear” (GOW1-R4) and “all the anger goes away” (MGW2-R9) when they reached the pitch. For some women, soccer made the worries abate entirely, so that they returned home without stress.

I used to have a lot of stress, but when I go play, I come back happy, and the thoughts are gone.

(MAW2-R11)

Although some women acknowledged that soccer provided only a temporary respite from stressful events, (“thoughts at home will never end” GOW1-R9), being part of the league helped women manage their daily stressors. 

#### 3.2.4. “*Exercising Has Improved My Health. I No Longer Fall Ill with Common Diseases.*” (MGW1-R7)

In the FGDs, women shared their various health concerns, the majority of which involved hypertension, headaches, sleeplessness, and being overweight. Women reported that soccer alleviated many of these concerns:

I will also add to that. For me I would lack sleep at times. I do not know if it was stress or what it may have been. I had to take Piriton [an analgesic] or paracetamol [acetaminophen], but now since I started ball, I don’t take medicine. When I go to sleep, I sleep as though I have taken Piriton. I mean it is Piriton. Let’s say it is like medicine now [laughter].

(LUW1-R12)

Women’s weight was a common theme in all of the FGDs and a number of them mentioned that the doctors at the clinics had advised them to lose weight. Soccer provided them with the means to do this:

High blood pressure was a major problem. The doctor used to encourage me to lose weight. I never used to eat a lot but the weight was constant. Since I joined the soccer team, my body has become more active and more fit. My pressure has decreased. I do not take the medicine anymore.

(MPW2-R9)

Playing soccer was generally viewed as a way to “help those with big bodies” (KIW1-R10), making women more fit both on and off the soccer pitch, and reducing the frequency of “mild illnesses” (JUW1-R3) and their need to take medication.

## 4. Discussion

This study used an explanatory sequential mixed methods approach to explore the health benefits of a soccer program for adult women in rural Kenya. Quantitative results suggested that being age 25 to 34 rather than 35–49 years of age, having one to two rather than 3 to 5 children, and living in a Maasai or mixed Maasai/settled community rather than a settled village were positively associated with good or excellent SRHS. The first two findings seem intuitively correct; younger women and women with fewer children may have fewer conditions that expose them to health risks or feel less burdened by the emotional and physical demands of daily life. Findings that women from Maasai communities have significantly greater odds of reporting good to excellent health compared to women from settled villages may reflect the very different physical and social worlds of the Maasai. Although research on this subject is scarce, a study of women in Kenya found that Maasai women had the highest levels of physical activity and, correspondingly, significantly lower levels of sedentary activity, compared to women from other tribes [25]. While all adult women in this setting have significant domestic responsibilities, Maasai women may be more “fit” due to the demands of an agro-pastoralist existence. In Maasai communities, women, once married, take responsibility for building and maintaining their dwellings and the milking of the herd as well as the many domestic duties associated with food provision, water collection, and child care [26].

Women who had completed secondary school or higher had lower odds of reporting good or excellent health than women who had no education. While it was beyond the scope of this study to determine the cause, there are a number of plausible explanations. Women with higher levels of education may be better informed about their state of health and therefore more aware that conditions such as hypertension and being overweight are associated with poor health [27]. Additionally, women who completed secondary school may have envisioned opportunities for themselves beyond the gender-normative roles of production and reproduction assigned to them, and this could be a source of unhappiness and poor emotional health. 

As hypothesized in this study, quantitative findings revealed that women’s participation in the NSL was associated with better self-reported health, and these findings were corroborated and described more fully in the qualitative findings. Women in FGDs, for example, reported that, since they began to play soccer, they felt “lighter” (a reference to having more energy and losing weight), stronger, and less tired and worried, both on and off the field. Extensive literature supports this link between sports and improved physical health. In the past two decades, sport has come to be an important strategy for improving health and well-being, particularly for its role in reducing the burden of preventable NCDs [28]. Research further suggests that physical activity is positively associated with mental health and may be an effective intervention to reduce the burden of depression [29]. Findings from previous work by the authors suggest that the social support from friends that women NSL members receive as members of the soccer program may function as a pathway to improved mental health [30]. Research has shown that social support can act as a buffer between stressful events and negative health effects [31]. Women may still have worries at home, but these worries seem to produce less stress and anxiety. 

Importantly, women assessed the health-benefits of soccer based upon the extent to which they are able to manage their daily responsibilities as wives and helpmates. Good health, as characterized by focus group participants, is when a woman’s chores are completed more easily and quickly, her husband is happy, and she does not need any medication to sleep or reduce aches and pains. Such metrics may be particularly salient for women in a setting in which biological measures of good health may be unavailable due to limited access to routine health care and its associated out-of-pocket expense.

In describing the benefits of playing soccer in terms of domestic responsibilities, women may also be justifying “leisure time” in a setting in which women rarely have time for physical activity outside of the occupational demands of home-keeping, child rearing, and food production. If the NSL is seen to help women complete their chores more quickly and easily and to improve partner dynamics at home, then playing soccer may be recast as a social good rather than simply as an activity that women do for themselves. 

### Limitations

This study has several limitations that should be noted. The NSL is part of a larger program that is widely endorsed by the participating communities. Community leaders, husbands, and family members accept that women spend some portion of time each week on Nikumbuke activities that yield benefits to households and communities. Under such circumstances, the setting aside of chores and responsibilities by women to engage in sport may not be viewed as idleness or time wasted in play but rather as another means by which to support the family. In the same vein, participants in the FGDs may have felt it socially desirable to speak positively about a program that they viewed as a source of this support. In addition, it is important to note that the soccer program was requested by women themselves rather than initiated by others for the purposes of improving health. Whether a stand-alone program or one that is brought to a community as part of a sport-for-development effort would be acceptable is not known. Importantly, despite quantitative and qualitative data in this study reporting the health benefits of the soccer program for adult women, these findings have not been validated by clinical markers. Research combining self-reported health effects with biodata would strengthen the evidence base for the utilization of soccer programs in other communities as health interventions.

## 5. Conclusions

Despite a national commitment to the provision of quality and accessible health care to all its citizens, Kenya continues to face challenges in operationalizing this vision, particularly in parts of the country like Lunga Lunga sub-county where clinics are often far from women’s homes and efforts to promote health are heavily focused on reducing maternal mortality, under-nutrition, and the burden of infectious diseases. Low-cost strategies that do not rely on the health care system and that can be introduced and locally sustained in rural areas may be particularly effective in stemming the rise in NCDs seen in Kenya and other LMICs. Soccer programs for adult women may be effective interventions in settings where access to health care is limited and where lack of opportunity to engage in physical aerobic activity increases women’s risks for poor health outcomes. Future research that uses clinical measures to assess the mental and physical effects of soccer on adult women’s health would further contribute to the evidence base for the expansion of the Nikumbuke program to other communities in Kenya and elsewhere.

## Figures and Tables

**Table 1:** Distribution of respondent attributes according to soccer participation and self-reported health status (*n* = 684).

Variable	Values			Self-Reported Health Status			
				Good to Excellent	Poor to Fair		
		*n*	%	%	%	χ^2^	%
Soccer membership						17.7	<0.001
	No	455	66.5	53.9	46.2		
	Yes	229	33.5	70.6	29.4		
	Missing	-	-				
Self-reported health status						-	-
	Fair or poor	277	40.5	-	-		
	Good or excellent	406	59.4	-	-		
	Missing	1	0.2				
Age						43.84	<0.001
	18–24 years	57	8.3	4.7	12.5		
	25–34 years	157	23.0	16.3	31.9		
	35–49 years	263	38.5	46.3	39.6		
	50–59 years	86	12.6	19.5	10.0		
	60 years or older	56	8.2	13.2	6.1		
	Missing	65	9.5				
Relationship status						13.97	0.001
	Married	536	78.4	75.0	81.2		
	Partnered but not married	31	4.5	2.5	5.9		
	Not in a relationship	114	16.7	22.5	12.9		
	Missing	3	0.4				
Children						13.87	0.003
	None	17	2.5	2.3	2.8		
	1–2	89	13.0	7.9	17.2		
	3–5	355	51.9	59.9	49.4		
	6 or more	202	29.5	30.0	30.6		
	Missing	21	3.1				
Education						16.35	0.003
	None	268	39.2	30.8	46.0		
	Some primary	209	30.6	34.4	28.8		
	Completed primary	129	18.9	22.7	16.5		
	Some secondary	22	3.2	3.7	3.0		
	Completed secondary or higher	46	6.7	8.4	5.8		
	Missing	10	1.5				
Work for wages in past 12 months						7.86	0.020
	No work for wages	481	70.3	73.1	72.3		
	Worked throughout year	65	9.5	6.3	12.2		
	Worked seasonally/occasionally	117	17.1	20.5	15.5		
	Missing	21	3.1				
Type of community						61.76	<0.001
	Maasai	95	13.9	2.5	21.7		
	Maasai/settled	47	6.9	4.0	8.9		
	Settled	471	68.9	82.3	59.6		
	Market town	71	10.4	11.2	9.9		
	Missing	-	-				
Health clinic in community						3.32	0.069
	No	515	75.3	57.2	42.8		
	Yes	156	22.8	65.4	34.6		
	Missing	13	1.9				
Pain in past 4 weeks interfered with normal work						69.49	.000
	Quite a bit/extremely	112	16.4	25.7	10.1		
	Moderately	89	13.0	21.4	7.4		
	Not at all/slightly	482	70.5	52.9	82.5		
	Missing	1	0.2				
Health in past 4 weeks limited moderate activities						25.04	0.000
	Limited a lot	50	7.3	11.4	4.7		
	Limited a little	260	38.0	45.4	33.9		
	Did not limit at all	365	53.4	43.2	61.4		
	Missing	9	1.3				
During past 4 weeks, did you have a lot of energy?						40.82	<0.001
	A good bit/all of time	395	57.8	28.6	13.8		
	Some of time	153	22.4	27.9	18.7		
	None/a little of time	135	19.7	43.5	67.5		
	Missing	1	0.2				
During past 4 weeks, have you felt downhearted and blue?						54.94	0.000
	A good bit/all of time	191	27.9	40.6	19.3		
	Some of time	123	18.0	22.1	15.3		
	None/a little of time	368	53.9	37.3	65.4		
	Missing	2	0.3				
Amount of time physical or emotional health interfered with social activities in past 4 weeks						30.92	<0.001
	A good bit/all of time	105	15.4	24.5	9.4		
	Some of time	73	10.7	12.04	9.9		
	None or a little of time	500	73.1	63.5	80.7		
	Missing	6	0.9				

**Table 2:** Factors associated with self-reported good or excellent health (*n* = 684).

	OR	*p*	95% CI
Soccer team participation (ref. Not in soccer)	1.66	0.021	1.08, 2.57
Type of community (ref. Settled village)			
Maasai	11.24	<0.001	4.49, 28.14
Maasai/Settled	2.75	0.020	1.18, 6.43
Market town	1.88	0.053	0.99. 3.55
Age (ref. 35 to 49 years)			
18 to 24	1.13	0.820	0.39, 3.25
25 to 34	1.86	0.028	0.07, 3.24
50 to 59	0.74	0.330	0.41, 1.35
60 and over	0.56	0.129	0.27, 1.18
Number of children (ref. 1 to 2)			
None	1.75	0.488	0.36, 8.59
3 to 5	2.37	0.021	1.14, 4.94
6 or more	1.47	0.099	0.93, 2.33
Relationship status (ref. Married)			
Partnered, not married	2.16	0.124	0.81, 5.77
Not in relationship	0.90	0.704	0.53, 1.53
Education (ref. None)			
Some primary	0.72	0.186	0.44, 1.17
Completed primary	0.60	0.085	0.33, 1.07
Some secondary	0.68	0.485	0.24, 1.98
Completed secondary or higher	0.37	0.028	0.15, 0.90
Worked for wages in past 12 months (ref. No wage work)			
Throughout year	2.11	0.052	0.99, 4.50
Seasonally	0.76	0.283	0.45, 1.26
Pain in past 4 weeks interfered w/work (ref. Slightly/not at all)			
Quite a bit/extremely	0.36	<0.001	0.21, 0.62
Moderately	0.24	<0.001	0.13, 0.43
Health limited moderate activities (ref. Not limited at all)			
Limited a lot	0.47	0.066	0.21,1.05
Limited a little	0.41	<0.001	0.27, 0.63
Has energy in past 4 weeks (ref. A good bit /all of the time)			
None of the time/a little of the time	0.99	0.970	0.59, 1.65
Some of the time	0.68	0.123	0.42, 1.10
Felt downhearted and blue in past 4 weeks (ref. None of time/a little of time)			
A good bit of time/all of time	0.54	0.009	0.34, 0.86
Some of time	0.70	0.172	0.41, 1.17
Physical/emotional health interfered with social activities (ref. None of time/a little of time)			
A good bit of time/all of time	0.60	0.071	0.35, 1.05
Some of time	0.83	0.588	0.43, 1.61

## Data Availability

The data presented in this study are available on request from the corresponding author. The data are not publicly available due to confidentiality concerns. Audio-visual data recording focus group discussions cannot be shared as permission was only received from participants to videotape sessions for the purposes of aiding the study team with transcription.

## References

[1] United Nations, Department of Economic and Social Affairs Sustainable Development Goal 3. https://sustainabledevelopment.un.org/sdg3.

[2] Nugent R., Bertram M.Y., Jan S., Niessen L.W., Sassi F., Jamison D.T., Pier E.G., Beaglehole R. (2018). Investing in non-communicable disease prevention and management to advance the Sustainable Development Goals. Lancet.

[3] Jaacks L.M., Slining M.M., Popkin B.M. (2015). Recent underweight and overweight trends by rural–urban residence among women in low-and middle-income countries. J. Nutr..

[4] NCD Risk Factor Collaboration (NCD-RisC)—Africa Working Group (2017). Trends in obesity and diabetes across Africa from 1980 to 2014: An analysis of pooled population-based studies. Int. J. Epidemiol..

[5] Goedecke J.H., Mtintsilana A., Dlamini S.N., Kengne A.P. (2017). Type 2 diabetes mellitus in African women. Diabetes Res. Clin. Pract..

[6] Ministry of Health Division of Communicable Diseases, Kenya National Bureau of Statistics, World Health Organization (2016). Kenya STEPwise Survey for Non Communicable Diseases Risk Factors 2015 Report 2016.

[7] World Health Organization (2015). UN, Kenyan Government Take Broad-Based Approach to Fighting NCDs. https://www.who.int/nmh/events/2014/kenya-ncd-prevention/en/.

[8] Republic of Kenya, Ministry of Health (2015). Kenya National Strategy for the Prevention and Control of Non-Communicable Diseases 2015–2020.

[9] Kruger H.S., Venter C.S., Vorster H.H., Margetts B.M. (2002). Physical inactivity is the major determinant of obesity in black women in the North West Province, South Africa: The THUSA study. Nutrition.

[10] Burnett C. (2002). Women, poverty and sport: A South African scenario. Women Sport Phys. Act. J..

[11] Abubakari A.-R., Bhopal R. (2008). Systematic review on the prevalence of diabetes, overweight/obesity and physical inactivity in Ghanaians and Nigerians. Public Health.

[12] Sobngwi E., Mbanya J.N., Unwin N.C., Kengne A.P., Fezeu L., Minkoulou E.M., Aspray T.J., Alberti K.G.M.M. (2002). Physical activity and its relationship with obesity, hypertension and diabetes in urban and rural Cameroon. Int. J. Obes..

[13] Micklesfield L.K., Lambert E.V., Hume D.J., Chantler S., Pienaar P.R., Dickie K., Goedecke J.H., Puoane T. (2013). Socio-cultural, environmental and behavioural determinants of obesity in black South African women. Cardiovasc. J. Afr..

[14] Puoane T., Matwa P., Hughes G., Bradley H.A. (2006). Socio-cultural factors influencing food consumption patterns in the black African population in an urban township in South Africa. Hum. Ecol..

[15] Creswell J.W., Creswell J.D. (2017). Research Design: Qualitative, Quantitative, and Mixed Methods Approaches.

[16] County Government of Kwale (2018). Kwale County Integrated Development Plan (2018–2022).

[17] Otieno G.A., Owenga J.A., Onguru D. (2020). Maternal Health Care Choices among Women in Lunga Lunga Sub County in Kwale County-Kenya. World J. Innov. Res..

[18] Republic of Kenya, Ministry of Health (2015). Kwale County, Health at a Glance. https://www.healthpolicyproject.com/pubs/291/Kwale%20County-FINAL.pdf.

[19] Alonso A., Langle de Paz T. (2019). Health by All Means: Women Turning Structural Violence into Peace and Wellbeing.

[20] Ware J.E., Sherbourne C.D. (1992). The MOS 36-item short-form health survey (SF-36): Conceptual framework and item selection. Med. Care.

[21] StataCorp (2019). Stata Statistical Software: Release 16.

[22] White I.R., Royston P., Wood A.M. (2011). Multiple imputation using chained equations: Issues and guidance for practice. Stat. Med..

[23] Rubin D.B. (1987). Multiple Imputation for Survey Nonresponse.

[24] Braun V., Clarke V. (2006). Using thematic analysis in psychology. Qual. Res. Psychol..

[25] Christensen D.L., Faurholt-Jepsen D., Boit M.K., Mwaniki D.L., Kilonzo B., Tetens I., Kiplamai F.K., Cheruiyot S.C., Friis H., Borch-Johnsen K. (2012). Cardiorespiratory fitness and physical activity in Luo, Kamba, and Maasai of rural Kenya. Am. J. Hum. Biol..

[26] Coast E. (2000). Maasai Demography.

[27] Aljassim N., Ostini R. (2020). Health literacy in rural and urban populations: A systematic review. Patient Educ. Couns..

[28] World Health Organization (2003). Health and Development through physical Activity and Sport.

[29] Saxena S., Van Ommeren M., Tang K.C., Armstrong T.P. (2005). Mental health benefits of physical activity. J. Mental Health.

[30] Barchi F., AbiNader M.A., Winter S.C., Obara L.M., Mbogo D., Thomas B.M., Ammerman B. “When You Mix with Your Friends, All the Stress Is Gone”: Adult Women’s Soccer as a Social Support Intervention in Rural Kenya.

[31] Cohen S., Wills T.A. (1985). Stress, social support, and the buffering hypothesis. Psychol. Bull..

